# The Inflammatory Response in Human Keratinocytes Exposed to Cinnamaldehyde Is Regulated by Nrf2

**DOI:** 10.3390/antiox11030575

**Published:** 2022-03-17

**Authors:** Romain Vallion, Kévin Hardonnière, Abderrahmane Bouredji, Marie-Hélène Damiens, Claudine Deloménie, Marc Pallardy, Pierre-Jacques Ferret, Saadia Kerdine-Römer

**Affiliations:** 1 Inserm, Inflammation Microbiome and Immunosurveillance, Université Paris-Saclay, 92290 Châtenay-Malabry, France; vallion@genoway.com (R.V.); kevin.hardonniere@universite-paris-saclay.fr (K.H.); a.bouredji@mines-stetienne.fr (A.B.); marie-helene.damiens@universit-paris-saclay.fr (M.-H.D.); marc.pallardy@universite-paris-saclay.fr (M.P.); 2 Safety Assessment Department, Pierre Fabre Dermo Cosmétique, 31000 Toulouse, France; pierre-jacques.ferret@pierre-fabre.com; 3 Inserm US31, CNRS UMS3679, Ingénierie et Plateformes au Service de l’Innovation Thérapeutique, Université Paris-Saclay, 92296 Châtenay-Malabry, France; claudine.delomenie@universite-paris-saclay.fr

**Keywords:** Nrf2, keratinocytes, inflammation, chemical sensitizer, Cinnamaldehyde

## Abstract

Keratinocytes (KC) play a crucial role in epidermal barrier function, notably through their metabolic activity and the detection of danger signals. Chemical sensitizers are known to activate the transcription factor nuclear factor (erythroid-derived 2)-like 2 (Nrf2), leading to cellular detoxification and suppressed proinflammatory cytokines such as IL-1β, a key cytokine in skin allergy. We investigated the role of Nrf2 in the control of the proinflammatory response in human KC following treatment with Cinnamaldehyde (CinA), a well-known skin sensitizer. We used the well-described human KC cell line KERTr exposed to CinA. Our results showed that 250 μM of CinA did not induce any Nrf2 accumulation but increased the expression of proinflammatory cytokines. In contrast, 100 μM of CinA induced a rapid accumulation of Nrf2, inhibited *IL-1β* transcription, and downregulated the zymosan-induced proinflammatory response. Moreover, Nrf2 knockdown KERTr cells (KERTr ko) showed an increase in proinflammatory cytokines. Since the inhibition of Nrf2 has been shown to alter cellular metabolism, we performed metabolomic and seahorse analyses. The results showed a decrease in mitochondrial metabolism following KERTr ko exposure to CinA 100 µM. In conclusion, the fate of Nrf2 controls proinflammatory cytokine production in KCs that could be linked to its capacity to preserve mitochondrial metabolism upon chemical sensitizer exposure.

## 1. Introduction

Skin is a highly complex organ forming a barrier that protects the body from water loss and environmental aggression (microorganisms, UV, and chemicals) [1]. These protections are ensured by the epidermis, the outer part of the skin, mainly composed of keratinocytes (KC) and some resident immune cells such as resident memory T cells and Langerhans cells (LC). KC are immunocompetent cells that can recognize immune danger signals and respond by secreting proinflammatory cytokines and chemokines, leading to skin inflammation [2].

The most common skin inflammation in humans is allergic contact dermatitis (ACD), characterized by eczematous dermatitis caused by repeated skin exposure to low molecular weight compounds, called haptens [3]. ACD is a T-cell-mediated inflammation that involves many cell types and immune factors. Among these different cell types, KC plays a crucial role in detecting haptens and initiating the ACD [4]. Haptens could be recognized as immune to danger signals by KC and dendritic cells (DC), provoking endogenous danger signaling by triggering reactive oxygen species (ROS) production. Indeed, skin pretreatment with antioxidants prevents hyaluronidase-mediated hyaluronic acid degradation, a crucial step in the sensitization phase of ACD [4,5].

In response to contact sensitizers (CS) behaving as haptens, KC released activated forms of IL-1β and IL-18 essential to initiate the sensitization phase [6,7]. IL-1β has multiple roles in ACD pathogenesis [7]. It is required for LC activation and migration from the skin to lymph nodes, for the recruitment of inflammatory cells to the skin, and also favors cluster of differentiation (CD) 4 T-cell polarization toward Th17 [8,9,10,11]. The essential role of IL-1β in ACD has already been shown in IL-1β knockout mice that failed to develop an ACD to trinitrochlorobenzene (TNCB) [10]. The NLRP3 inflammasome is required to transform inactive pro-IL-1β into active IL-1β, and NLRP3-deficient mice had reduced ear swelling in response to TNCB [6]. TNCB-induced ACD was lower in IL-1R-deficient mice, in which IL-1β signaling is missing [6]. Therefore, IL-1β regulation and function are crucial for ACD development. In response to CS, a transcriptomic analysis of KC revealed a set of genes regulated explicitly by CS [12]. Other in vitro studies have identified a proteomic change in KC associated with filaggrin deficiency found in the skin of atopic dermatitis patients [13,14].

Skin inflammation induced by CS has been shown to be controlled by nuclear factor (erythroid-derived 2)-like 2 (Nrf2). Indeed, our group and others have previously demonstrated that Nrf2 regulates ACD in response to moderate or strong sensitizers [15,16]. Cinnamaldehyde (CinA), a major compound in cinnamon, is described as having antimicrobial, anti-inflammatory, and antitumoral effects [17,18]. However, it is also known to induce skin allergies [19]. It is a moderate CS that strongly activates Nrf2 in dendritic cells and induces an allergic inflammatory response only in Nrf2-deficient mice, indicating the essential role of Nrf2 [15,20]. However, its role in KCs remains to be explained.

Nrf2 has been initially described for its role in the antioxidant response. At a steady state, Nrf2 is linked to its negative regulator kelch-like ECH-associated protein 1 (Keap 1), which leads to Nrf2 ubiquitinoylation, and then favors its degradation by the proteasome [21]. Cellular exposure to oxidative or electrophilic stresses, such as CS-oxidizing cysteine residues of Keap1, alter its conformation, resulting in Nrf2 release, allowing its nuclear translocation. Once in the nucleus, Nrf2 binds to antioxidant response element (ARE) regulatory sequences among cytoprotective genes, such as *NQO1* (NAD(P)H quinone oxidoreductase 1) and *HO-1* (heme oxygenase-1) [22]. More recently, the role of Nrf2 in the control of inflammation has been documented in different immune cell types and inflammatory diseases [23]. The anti-inflammatory role of Nrf2 is both direct and indirect. Nrf2 can bind to DNA close to proinflammatory cytokines genes such as IL-1α, IL-1β, and IL-6 and, therefore, block RNA polymerase recruitment and gene transcription [24,25]. The indirect action of Nrf2 goes through Keap-1, leading to NF-кB inactivation, the main transcription factor involved in the expression of many proinflammatory cytokines [26].

Nrf2 has also been implicated in metabolism regulation, since it participates in the expression of many proteins involved in cellular metabolism pathways, such as glycolysis, pentose phosphate, nucleotide, and lipid metabolism. Therefore, Nrf2 activity can modify the overall cellular metabolism and intracellular metabolites [27,28]. Moreover, the Nrf2-induced HO-1 protein participates in heme catabolism, also altering the metabolism [29]. As previously shown in most immune cell types, a metabolic reprogramming occurs during their activation, and a modification of the intracellular metabolite compositions has been related to this inflammatory status [30,31,32]. These observations indicate that anti-inflammatory properties of Nrf2 could also be linked and/or regulated to metabolic changes.

We hypothesize that Nrf2 may prevent or attenuate the proinflammatory response in KC through a balance involving cytokines, chemokines, and cellular metabolite levels. This work investigates the role of Nrf2 in KC activation in response to CinA using a two-dimensional model of KC, the human KERTr cell line, exposed to two concentrations (100 µM and 250 µM) chosen based on Nrf2 accumulation. The ultimate objective is to decipher the link of Nrf2 in the activation of KCs in response to allergenic molecules and its connection with the metabolic profile of KCs.

## 2. Materials and Methods

### 2.1. Cell Culture and Treatment

All experiments were performed using CCD 1106 KERTr, a human primary KC-derived cell line (LGC Standards, Molsheim, France). As recommended by the manufacturer, KERTr cells were cultured at 37 °C, and 5% of CO_2_ in a keratinocyte–SFM serum-free medium (Gibco^TM^, Thermo Fisher Scientific, Illkirch, France) supplemented with 0.05 mg/mL of bovine pituitary extract (Gibco^TM^, Thermo Fisher Scientific, Illkirch, France), 35 ng/mL of epithelial growth factor (EGF; Miltenyi Biotec, Paris, France), 100 U/mL of penicillin, and 100 µg/mL of streptomycin (Gibco^TM^, Thermo Fisher Scientific, Illkirch, France). One day before treatment, cells were plated at a density of 2 × 10^5^ cells/cm^2^ with 1 mL per 10^6^ of cells culture medium without EGF. Cells were treated with 0.1% dimethyl sulfoxide (DMSO) as a control of CinA (Sigma-Aldrich, L’lsle D’Abeau Chesnes, France) at the indicated concentration and for the indicated time. For some experiments, when indicated, the cells were treated with 20 µg/mL of Zymosan A (Sigma-Aldrich, France).

### 2.2. Invalidation of Nrf2

Nrf2 expression has been invalidated using shRNA. The expression plasmids for Nrf2 shRNA (OriGene Technologies GmbH, Herford, Germany) were transferred into the KERTr cell using lentiviral transduction. A scrambled shRNA was used as a control. Briefly, 2 × 10^6^ cells at 50% confluency were incubated overnight with 10 µg/mL of polybrene and 200 µg/mL of viral particles. Control cells (sh Sc) and invalidated cells for Nrf2 (sh Nrf2) were sorted on a FACS Aria (BD Biosciences, Le Pont de Claix, France) based on the GFP expression.

### 2.3. Cell Toxicity

KERTr cells were seeded at 4 × 10^5^ cells/well in a 24-well plate and treated the next day with increasing concentrations of CinA (5–1000 µM) or 0.1% of DMSO for 24 h. Then, cells were harvested with Trypsin–0.25% EDTA (Gibco^TM^, Thermo Fisher Scientific, Illkirch, France) and stained with one microgram of propidium iodide (Thermo Fisher Scientific, Illkirch, France). Cell fluorescence was acquired using the Attune Nxt (Thermo Fisher Scientific) and analyzed with FlowJo software version 8.0.2.

### 2.4. Western Blot Analysis

After stimulation, the wells were washed 2 times with cold Phosphate-buffered saline (PBS), and the cells were lysed with lysis buffer (20-mM Tris (pH 7.4), 137-mM NaCl, 2-mM Ethylenediaminetetraacetic acid (EDTA) (pH 7.4), 2-mM sodium pyrophosphate, 1% Triton, 10% glycerol, 1-mM PMSF, 1-mM Na_3_VO_4_, 25-mM β-glycerophosphate, 10-μg/mL aprotinin, 10-μg/mL leupeptin, and 100-μg/mL pepstatin). After centrifugation at 11,000× *g* for 20 min at 4 °C, 40 μg of denatured protein were loaded onto 10% of sodium dodecyl sulfate- polyacrylamide gel electrophoresis (SDS-PAGE) gels (TGX Stain-Free FastCast^TM^, Bio-Rad^®^, Marnes la Coquette, France) and transferred to polyvinylidene fluoride (PVDF) membranes, which were successively blocked, incubated with primary antibodies anti-Nrf2 (1/1000e, 16396-1-AP, Proteintech, Manchester, United Kingdom), anti-HO-1 (1/1000e, ab13248, Abcam, Paris, France), anti-NQO1 (1/1000e, ab28947, Abcam, Paris, France), and then with secondary antibodies conjugated to Horseradish peroxidase (HRP). Immunoreactive bands were detected by chemiluminescence using the ChemiDoc XRS+ System (Bio-Rad Laboratories, Marnes la Coquette, France). Bands were quantified with Image Lab software and normalized with the total protein loaded [33].

### 2.5. Quantitative Reverse Transcription-Polymerase Chain Reaction

According to the manufacturer’s instructions, the total RNA was extracted with a PureLink™ RNA Mini kit (Invitrogen^®^, Thermo Fisher Scientific, Illkirch, France). cDNA was synthesized from 500 ng of total RNA with 2.5 µM of oligo(DT) (Promega^®^, Charbonnières-Les-Bains, France), 0.5 mM of dNTP (MP Biomedicals^®^, Illkirch, France), 1 U/µL of SuperScript™ IV Reverse Transcriptase (Invitrogen^®^, Thermo Fisher Scientific, Illkirch, France), 1 U/µL of RNasine (Promega^®^, Charbonnières-Les-Bains, France), 5 mM of DTT (Dithiothréitol, Invitrogen^®^, Thermo Fisher Scientific, Illkirch, France), and 1X Reverse Transcriptase buffer (Invitrogen^®^, Thermo Fisher Scientific, Illkirch, France) to a final volume of 20 µL. qPCR was performed with SYBR^TM^ Green technology on a CFX384 system (Bio-Rad, Marnes-la-Coquette, France). Each reaction mix consisted of 5 ng of cDNA, 0.5 μM of each forward and reverse primer, and SSo Advanced Supermix (Bio-Rad, Marnes-la-Coquette, France) in a total reaction volume of 10 μL. The following specific primers were used: After 30 s at 95 °C for Sso7dfusion polymerase activation, amplification was allowed to proceed for 44 cycles, each consisting of denaturation at 95 °C for 5 s and annealing/extension at 60 °C for 5 s. Eightfold serial dilutions of mixed cDNA (from different samples) were analyzed for each target gene, enabling us to construct linear standard curves from which the efficiency (E) of each PCR run was evaluated. SYBR green fluorescence was detected at the end of each elongation cycle; after which, a melting curve was constructed to confirm the specificity of the PCR products. Quantification was performed with CFX Manager Software version 3.1 (Bio-Rad, Marnes-la-Coquette, France), and data were analyzed by the ΔΔCt method. Ratios were calculated as the geometric mean of (1 + E)^(−ΔΔCt), where E is the efficiency, and ΔΔCt is the target gene expression of treated cells compared with normal levels in untreated cells, with correction for the expression of the reference genes *ACTIN-β* and *GAPDH*. RT-qPCR results are expressed as the fold factor increase (i.e., ratio of (1 + E) ^(−ΔΔCt) of treated cells/(1 + E) ^(−ΔΔCt) of untreated cells).

### 2.6. Cytokines Production Assessment

The level of proinflammatory cytokines (IL-1α, IL-1β, IL-6, IL-8, IL-23, TNF-α, and TSLP) was measured in the supernatants of cells stimulated for 24 h by CinA, Zymosan, or CinA and zymosan by the Meso Scale Discovery (Meso Scale Diagnostics, Rockville, MD, USA) multiplex assay, according to the manufacturer’s instructions.

### 2.7. Targeted Liquid Chromatography–Mass Spectrometry Metabolites Analyses

After stimulation by CinA, metabolites extraction was performed on 7.5 × 10^5^ cells with a solution of 50% methanol, 30% acetonitrile, and 20% water. After the addition of extraction solution (1 mL per 2 × 10^6^ cells), samples were vortexed for 5 min at 4 °C and then centrifuged at 16,000× *g* for 15 min at 4 °C. The supernatants were collected and stored at −80 °C until analysis. Liquid chromatography–mass spectrometry analyses were conducted on a QExactive Plus Orbitrap mass spectrometer equipped with an Ion Max source and a HESI II probe and coupled to a Dionex UltiMate 3000 UPLC system (Thermo Fisher Scientific). The metabolites were detected across a mass range of 75–1000 *m/z* at a resolution of 35,000 (at 200 *m/z*), and data were acquired with Thermo Xcalibur software version 4.3 (Thermo Fisher Scientific). The peak areas of the metabolites were determined using Thermo TraceFinder software (Thermo Fisher Scientific), identified by the exact mass of each singly charged ion and by the known retention time on the high performance liquid chromatography (HPLC) column.

### 2.8. Analysis of Oxygen Consumption and Extracellular Acidification Rates

To measure the cell metabolism in intact KERTr cells, an XF96 Extracellular Flux Analyzer (Agilent Technologies, Santa Clara, CA, USA) was used with the “ATP rate assay kit” to measure multiple parameters, including the oxygen consumption rate (OCR), extracellular acidification rate (ECAR), and total ATP production by quantifying the ATP production rate from both glycolytic and mitochondrial pathways. The optimal seeding density for the KERTr cell line was determined to be 30,000 cells per well. A day prior to the experiment, a seahorse sensor cartridge was hydrated and incubated in a non-CO2 incubator at 37 °C. Two hours before the experiment, the sensor cartridge was loaded with the seahorse calibrant. On the day of the experiment, seahorse XF DMEM (pH = 7.4) was supplemented with 1-mM pyruvate, 2-mM glutamine, and 10-mM glucose (Agilent, Technologies, Santa Clara, CA, USA). The Keratinocyte SFM-free medium was then replaced with 180 µL of seahorse XF base medium, and the cells were incubated in a non-CO_2_ incubator at 37 °C for 1 h. Oligomycin and Rotenone/antimycin (Agilent Technologies, Santa Clara, CA, USA) were prepared in the seahorse medium to achieve final concentrations of 1.5 μM and 0.5 μM, respectively, when injected. From these stock solutions, 20 μL and 22 μL of the Oligomycin and rotenone/antimycin mix were, respectively, loaded into the drug delivery ports A and B of the sensor cartridge and loaded into the seahorse XF analyzer to calibrate for 30 min. The calibration plate was then replaced with the cell culture plate, and OCR and ECAR were monitored for 1 h following the sequential injection of Oligomycin and rotenone/antimycin. According to the manufacturer’s instructions (Agilent Technologies, Santa Clara, CA, USA), acquisition and analyses were performed using the wave software. Data were normalized to the protein content by performing a BCA assay following the seahorse experiment.

The rate of ATP production from oxidative phosphorylation and glycolysis was then analyzed by monitoring the OCR and ECAR, respectively [34,35].

### 2.9. Statistical Analyses

Nonparametric analyses were performed using GraphPad Prism software: a Mann–Whitney test was used to compare two independent groups. Data are expressed as the mean ± SEM. Data are considered statistically different when the *p*-value < 0.05.

## 3. Results

### 3.1. Nrf2 Pathway Activation in Response to CinA

Cytotoxicity experiments using propidium iodide staining and flow cytometry allowed to determine two concentrations of CinA: a concentration of CinA leading to cell viability above 70%, 100 µM, and a concentration of CinA above 50% of cell viability (CV 50%), 250 µM (Figure S1).

**Figure 1:**
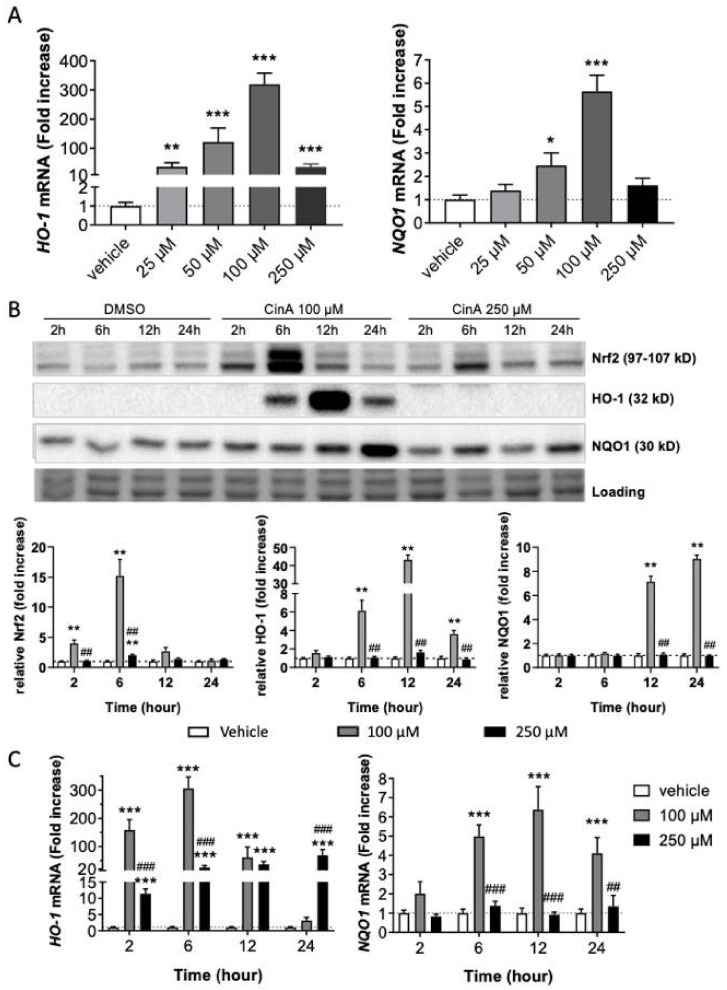
Nrf2 pathway activation in response to CinA. KC were exposed to different concentrations (25–250 µM) for 2–24 h. (**A**) mRNA level of HO-1 and NQO1 measured by RT-qPCR after 6 h of exposure to different concentrations (25, 50, 100, and 250 µM) of CinA and DMSO 0.1% as the vehicle control. (**B**) Western blot and relative quantification of Nrf2, HO-1, and NQO1 after exposure to CinA or DMSO 0.1% (2, 6, 12, and 24 h). Representative of 6 independent experiments. (**C**) mRNA level of HO-1 and NQO1 measured by RT-qPCR after exposure to 100 and 250 µM of CinA and DMSO 0.1% as the vehicle control (2–24 h). Data represent the results of 5–16 independent experiments and are expressed as the mean ± SEM. (**A**–**C**) * Represents the statistical difference between CinA and vehicle-treated cells. (B,C), # Difference between 100 µM and 250 µM-treated cells. *, *p*-value < 0.05, **, ## *p*-value < 0.01, and ***, ### *p*-value < 0.001 (Mann–Whitney test).

Then, we studied the Nrf2 pathway at different concentrations under the CV 50% (25, 50, 100, and 250 µM). Analysis of the mRNA expression of two Nrf2-target genes (*HO-1* and *NQO1*) following 6 h of CinA exposure showed an increase of their expression in a concentration-dependent manner (Figure 1A). The maximal mRNA expression was found at 100-µM CinA (Figure 1A). The highest concentration tested of CinA (250 µM) failed to induce *NQO1* mRNA, and *HO-1* mRNA expression was significantly reduced when compared to 100 µM (36.5 vs. 319.4-fold increase; Figure 1A). Moreover, we showed that CinA 100 µM induced an accumulation of Nrf2, induced HO-1, and augmented NQO1 at the protein level 6 h post-exposure (Figure 1B). In contrast, CinA 250 µM failed to accumulate Nrf2 and did not induce any expression of the HO-1 and NQO1 proteins (Figure 1B).

Regarding the levels of the mRNA target genes, kinetic analyses showed that CinA 100 µM induced a greater amount of *HO-1* compared to CinA 250 µM. For the 250-µM concentration, the amount of *HO-1* mRNA was induced in a lower quantity compared to CinA 100 µM. This induction was maintained all along the kinetics. For *NQO1* mRNA expression, only CinA 100 µM was able to induce its transcription (Figure 1C).

Therefore, our results demonstrate a differential effect in Nrf2 accumulation and activation depending on the CinA concentration, with a weaker induction of the Nrf2 pathway for the highest concentration (250 µM).

### 3.2. Inflammatory Response in KC Is Dependent on CinA Concentration

**Figure 2:**
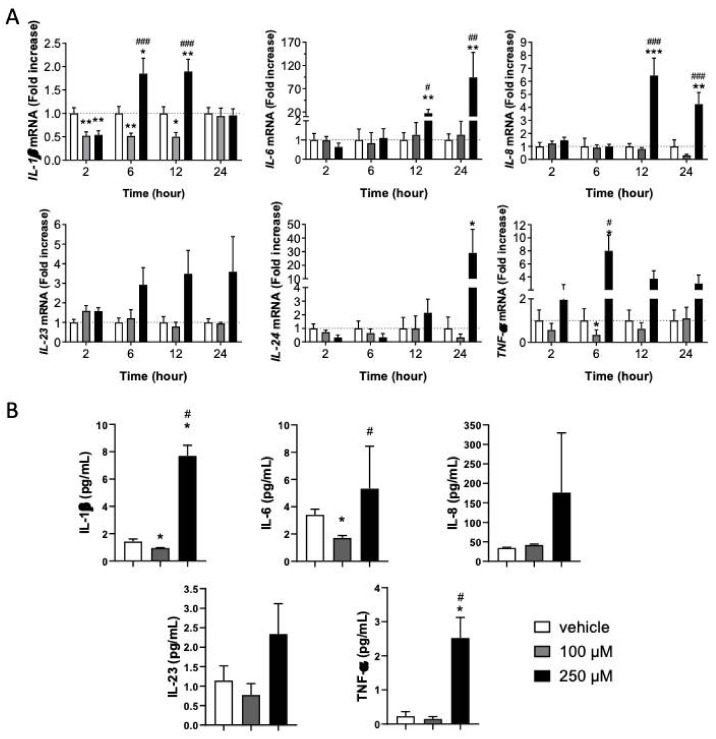
KC’s inflammatory response is dependent on the CinA concentration. KCs were exposed to 100 µM or 250 µM of CinA from 2 to 24 h. (**A**) The mRNA level of inflammatory cytokines IL-1β, IL-6, IL-8, IL23, IL-24, and TNF-α determined by RT-qPCR after 2–24 h of exposure to 100 and 250 µM of CinA and DMSO 0.1% as the vehicle control. (**B**) The inflammatory cytokines level was determined by Meso Scaled Discovery technology in the supernatant of KERTr after 24 h of exposure to 100 and 250 µM of CinA. Data represent the results of 3–14 independent experiments and are expressed as the mean ± SEM. (**A**,**B**) * Represents the statistical difference between CinA and vehicle-treated cells, and # represents the statistical difference between 100 µM and 250 µM-treated cells. *,# *p*-value < 0.05, **,## *p*-value < 0.01, and ***,### *p*-value < 0.001 (Mann–Whitney test).

Since Nrf2 has been shown to regulate the inflammatory response, especially the levels of IL-1β, TNF-α, and IL-6, we studied the inflammatory status of KC in response to both concentrations of CinA [25]. The mRNA levels of different cytokines *(IL-1β, IL-6, IL-8, IL-23, IL-24,* and *TNF-α*) by RT-qPCR have been assessed (Figure 2A).

Compared to untreated KC, the lower CinA concentration (100 µM) downregulated *IL-1β* and *TNF-α* mRNAs. Both were decreased for at least 12 h. Among the cytokines tested, none were upregulated by CinA 100 µM. In contrast, CinA 250 µM induced *IL-1β* 6 h post-exposure (Figure 2A). Other inflammatory cytokines, such as *IL-6*, *IL-8*, and *TNF-α,* were mainly enhanced.

We then measured the level of cytokine released after 24 h of exposure to CinA by MSD technology. CinA 100 µM significantly decreased the amount of IL-6 in the supernatant, while other tested cytokines were non-significantly reduced (Figure 2B). In contrast, CinA 250 µM significantly increased the amount of all proinflammatory cytokines released by KC (Figure 2B).

Altogether, our results revealed that the low concentration of CinA did not induce any proinflammatory cytokine gene transcriptions, whereas the highest concentration led to an inflammatory state in KCs by modulating both the transcription and secretion of proinflammatory cytokines.

### 3.3. Concentration of CinA inducing Nrf2 Controls Zymosan-Induced KC Inflammation

**Figure 3:**
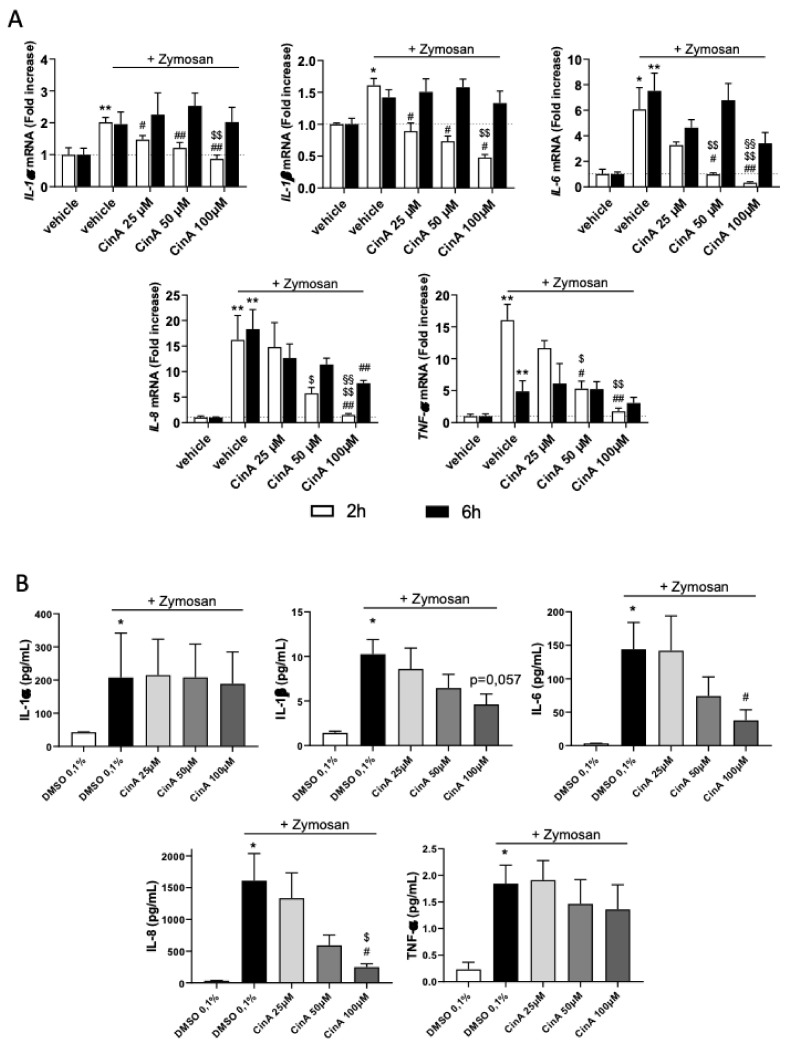
Low concentration of CinA controls zymosan-induced KC’s inflammatory response. KCs were exposed to different concentrations (25–100 µM) of CinA at the same time of the zymosan A (20 µg/mL) stimulation for 2, 6, and 24 h. (**A**) The mRNA level of inflammatory cytokines IL-1α, IL-1β, IL-6, IL-8, and TNF-α determined by RT-qPCR after 2 and 6 h of exposure to zymosan ± CinA (25, 50, or 100 µM) or DMSO 0.1% as the control. (**B**) Inflammatory cytokine levels determined by Meso Scaled Discovery technology in the supernatant of KERTr after 24 h of exposure to zymosan ± CinA (25, 50, or 100 µM). Data represent the results of 4–6 independent experiments and are expressed as the mean ± SEM. (**A**,**B**) * Represents the statistical difference between zymosan and vehicle-treated cells, # represents the statistical difference between zymosan ± CinA and zymosan-treated cells, $ represents the statistical difference between 50 or 100 µM and 25 µM of CinA and § represents statistical difference between 100 µM and 50 µM of CinA. *, #, $ *p*-value < 0.05 and **, ##, $$, §§ *p*-value < 0.01.

As CinA 100 µM induced an anti-inflammatory phenotype of KC, we then addressed the effect of this concentration of CinA under a proinflammatory environment. The KCs were exposed to Zymosan, a TLR2 agonist known to induce proinflammatory cytokines, and were treated or not with CinA (25–100 µM). A co-stimulation of KC by zymosan and CinA did not modify the antioxidant response induced by CinA (Figure S2), since HO-1 and NQO1 mRNAs were equally expressed. While zymosan alone increased the mRNA of *IL-1α*, *IL-1β*, *IL-6*, *IL-8*, and *TNF-α*, co-stimulation with zymosan and CinA resulted in lower mRNA levels of these five cytokines (Figure 3A). Moreover, the CinA effect was concentration-dependent, with a higher effect at 100 µM (Figure 3A) compared to the lowest concentrations tested (25 and 50 µM).

The level of cytokines in the supernatant measured by electroluminescence showed that CinA 100 µM significantly decreased the zymosan-induced IL-8 release, whereas IL-1β and IL-6 were also decreased but not significantly (Figure 3B).

### 3.4. The Inflammatory Response in KC Induced by CinA Is Nrf2-Dependent

Based on the literature showing the anti-inflammatory role of Nrf2 and the effect of CinA 100 µM in the control of some proinflammatory cytokines (Figure 2 and Figure 3), we next hypothesized that Nrf2 activation could be involved in the control of the inflammatory response upon CinA exposure [25]. To strengthen this idea, the analysis of the *IL-1β* mRNA level and Nrf2 accumulation at early time points revealed that these two phenomena were concomitant and occurred very rapidly following the 100-µM CinA stimulation (Figure S3).

**Figure 4:**
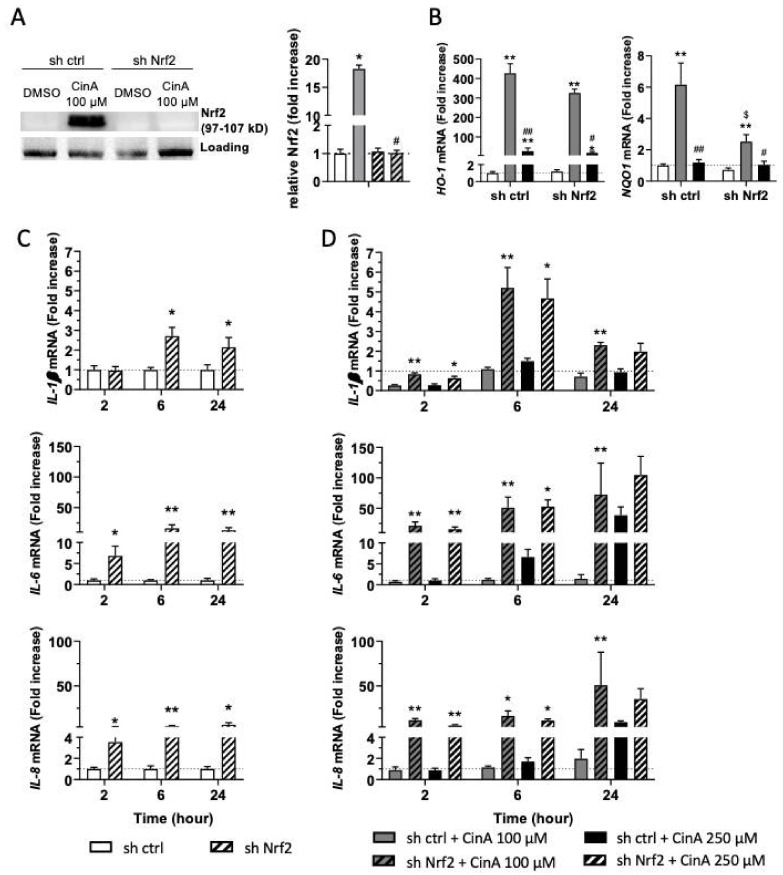
The control of the inflammatory in response to CinA is Nrf2-dependent. KCs were invalidated for Nrf2 by transduction with lentiviral particles to express a short-hairpin RNA targeting Nrf2 (sh Nrf2) or a scrambled short-hairpin (sh ctrl) as the control cells, and the cells were exposed to 100 or 250 µM of CinA from 2 to 24 h. (**A**) Western blot and relative quantification of Nrf2 after exposure to CinA or DMSO 0.1% as the vehicle control for 6 h. Representative of 4 independent experiments. (**B**) mRNA levels of HO-1 and NQO1 measured by RT-qPCR after 6 h of exposure to 100 or 250 µM of CinA and DMSO 0.1% as the vehicle control. (**C**) mRNA level of inflammatory cytokines IL-1β, IL-6, and IL-8 determined by RT-qPCR after 2, 6, and 24 h of culture without any stimulation. (**D**) mRNA levels of inflammatory cytokines IL-1β, IL-6, and IL-8 determined by RT-qPCR after 2, 6, and 24 h of exposure to 100 or 250 µM of CinA. Data represent the results of 5 independent experiments and are expressed as the mean ± SEM. (**A**,**B**) * Represents the statistical difference between CinA and vehicle-treated cells, # represents the statistical difference between 100 µM and 250 µM-treated cells, and $ represents the statistical difference between sh ctrl and sh Nrf2 cells. (**C**,**D**) * Represents the statistical difference between sh ctrl and sh Nrf2 cells. *, # *p*-value < 0.05 and **, ## *p*-value < 0.01 (Mann–Whitney test).

We then generate KERTr cells invalidated for Nrf2 expression using a ShRNA (sh Nrf2) (Figure 4A). The downregulation of Nrf2 expression had no effect on CinA cytotoxicity (Figure S4).

In sh Nrf2 cells, CinA 100 µM failed to induce *NQO1* mRNA transcription, while *HO-1* was only slightly increased (Figure 4B). At a steady state, the absence of Nrf2 in KC increased the basal levels of *IL-1β*, *IL-6,* and *IL-8* transcription (Figure 4C). In response to CinA 100 µM, the downregulation of *IL-1β* was abrogated in Nrf2-deficient cells, and transcription and secretion of the cytokines previously tested were significantly increased. For KC stimulated with 250-µM CinA, proinflammatory cytokines were increased at 2 and 6 h in the absence of Nrf2 compared to control cells. Interestingly, the mRNA levels of proinflammatory cytokines rise to the same level in Nrf2-deficient cells in response to both 100 µM and 250 µM of CinA (Figure 4D).

Altogether, our results further confirmed the critical role of Nrf2 to counteract CinA-induced proinflammatory cytokine transcription and secretion.

### 3.5. Concentration of CinA inducing Nrf2 Leads to Nrf2-Dependent Metabolic Reprogramming

**Figure 5:**
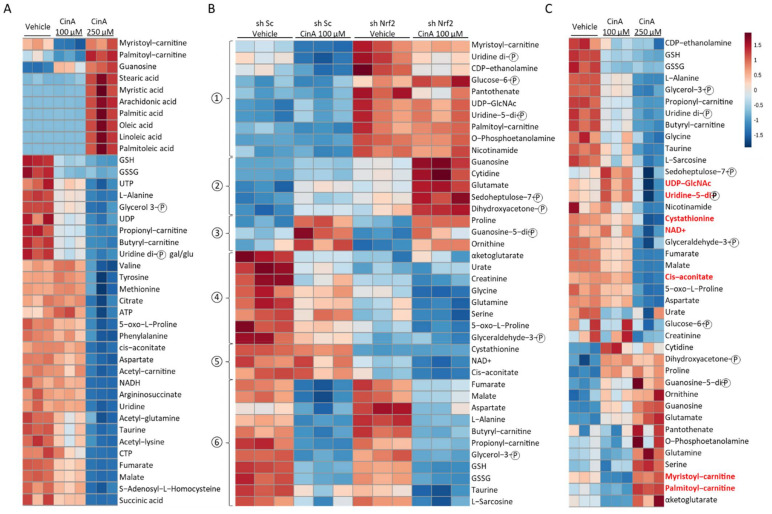
CinA and Nrf2 modify intracellular metabolites profiles in KCs. KCs were invalidated for Nrf2 by transduction with lentiviral particles to express a short-hairpin RNA targeting Nrf2 (sh Nrf2) or a scrambled short-hairpin (sh ctrl) as the control cells, and the cells were exposed to 100 or 250 µM of CinA for 1 h, followed by a metabolomic analysis by mass spectrometry. (**A**) Heatmap representing the 40 highest and most significant *p*-values from the comparison of sh ctrl cells exposed to CinA (100 or 250 µM) and DMSO 0.1% as the control. (**B**) Heatmap representing the 40 highest and most significant *p*-values from the comparison of sh ctrl and sh Nrf2 cells exposed to 100 µM of CinA or DMSO 0.1% as the control. (**C**) Heatmap representing the same metabolites as panel B for the comparison of sh ctrl cells exposed to CinA (100 or 250 µM) and DMSO 0.1% as the control. Data represent the results of 3 independent experiments.

As previously shown in most immune cell types, a metabolic reprogramming occurs during their activation, and a modification of the intracellular metabolite composition has been related to this inflammatory status [30,31,32]. Since Nrf2 is known to regulate the expression of many proteins implicated in cellular metabolism pathways, such as glycolysis (GLUT1, hexokinase 1); the pentose phosphate pathway (glucose-6-phosphate dehydrogenase); nucleotide biosynthesis (phosphoglycerate dehydrogenase); and lipid metabolism (ACOX1, ACOX2, CPT1, and CPT2), thus strongly impacting the overall composition of intracellular metabolites [27,28], we next performed a metabolomic analysis by mass spectrometry in the KERTr cell line following CinA stimulation (Table S1). The analysis of the 40 highest and significant *p*-values (*p*-values < 0.05) among the 115 metabolites analyzed revealed that the CinA 100 µM-treated cells metabolic profile was comparable to the control, except for a few metabolites, including guanosine, myristoyl-carnitine, and palmitoyl-carnitine (Figure 5A). In CinA 250 µM-treated cells, fatty acids were overrepresented, while other metabolites were dramatically decreased (Figure 5A). To address the role of Nrf2 in the modifications induced by CinA, we then compared the effect of CinA 100 µM in the sh Sc and sh Nrf2 KERTr cell lines. The 40 highest and significant *p*-values among the 115 metabolites analyzed are shown in Figure 5B. Based on the profile of the modifications identified on the heatmap, we were able to classify six different groups (Figure 5B). The analysis identified metabolites regulated in a Nrf2-dependent manner (groups 1, 2, 4, and 5) or independently (groups 3 and 6). Among the Nrf2-regulated groups, we were able to discriminate three groups regarding the effect of CinA. The compositions of groups 1 and 5 were not modified, whereas group 2 and group 4 metabolites were, respectively, upregulated and downregulated following CinA stimulation. Regarding metabolites not regulated by Nrf2, CinA increased some in group 3 and decreased others in group 6 (Figure 5B). Among the same 40 metabolites previously selected, seven metabolites regulated in response to CinA 250 µM (UDP-N-acetylglucosamine, Uridine 5′-diphospho-N-acetylgalactosamine, cystathionine, NAD^+^, Cis-aconitate, myristoyl-carnitine, and palmitoyl-carnitine) were similarly controlled in Nrf2 absence (Figure 5C).

**Figure 6:**
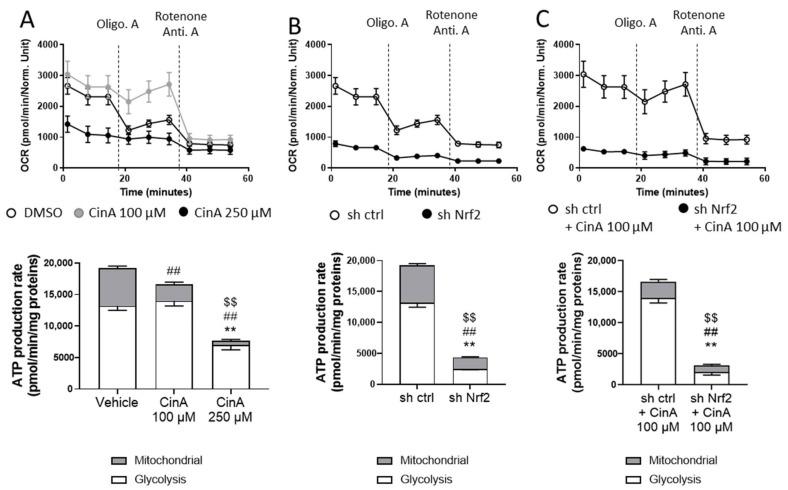
CinA and Nrf2 drive metabolic reprograming in KCs. KCs were invalidated for Nrf2 by transduction with lentiviral particles to express a short-hairpin RNA targeting Nrf2 (sh Nrf2) or a scrambled short-hairpin (sh ctrl) as the control cells, and the cells were exposed to 100 or 250 µM of CinA for 1 h, followed by the seahorse analysis. (**A**) Oxygen consumption rate (OCR) and ATP production of sh ctrl cells exposed to CinA (100 or 250 µM) and DMSO 0.1% as the control. (**B**) OCR and ATP production of unstimulated sh ctrl and sh Nrf2. (**C**) OCR and ATP production of sh ctrl and sh Nrf2 cells exposed to 100 µM of CinA and DMSO 0.1% as the control. Data represent the results of 1 experiment with 8 replicates. (**A**) *, #, and $ represent statistical differences, between the vehicle and CinA-treated cells for glycolytic, mitochondrial, and total ATP production respectively. (**B**,**C**) *, #, and $ represent statistical differences between sh Sc and sh Nrf2 cells for glycolytic, mitochondrial, and total ATP production, respectively. **, ##, $$ *p*-value < 0.01 (Mann–Whitney test).

Since the metabolomic analyses revealed different profiles regarding the concentration of CinA and Nrf2 expression, energetic metabolism using seahorse technology has been studied. We used the ATP rate assay kit to determine the oxygen consumption rate (OCR) and extracellular acidification rate (ECAR), allowing us to determine the respective amounts of ATP synthetized by glycolysis and mitochondria. OCR, which is related to mitochondrial activity and oxidative phosphorylation (OXPHOS), was slightly increased in response to CinA 100 µM compared to the vehicle cells. After the Oligomycin A treatment, this difference markedly increased, since the OCR dropped only for vehicle-treated cells. For CinA 250 µM, the level of OCR was very low (Figure 6A, upper panel). The total ATP production was not statistically different between these two groups, but CinA 100 µM-treated cells relied more on glycolysis to produce ATP (Figure 6A, lower panel). For ECAR, which is related to glycolysis, it was not affected by CinA 100 µM but greatly reduced in response to CinA 250 µM (Figure S5a). At a steady state, the sh Nrf2 was responsible for a very important decrease of ATP synthesis, with a drop in both OCR and ECAR. (Figure 6B and Figure S5b). The treatment of sh Nrf2 cells by CinA 100 µM did not show any metabolic switch, as for Nrf2-competent cells, further indicating that, as previously hypothesized, the protective effects of CinA 100 µM are most likely related to Nrf2 activity (Figure 6C and Figure S5c).

## 4. Discussion

In the skin, a gradient of Nrf2 expression has been described in the epidermis with a higher expression and activity in the suprabasal layer compared to the basal layer [36]. The protective role of Nrf2 has been demonstrated in different in vivo models of inflammation, such as ACD, autoimmune encephalomyelitis, or arthritis. In this context, pharmacological activation of the Nrf2 pathway has been shown to be a promising approach in both animal models and humans for regulating the inflammatory response induced by chemicals, neurodegeneration, or arthritis [15,23,37,38]. Among these activators, CinA, successfully used to mitigate gastric inflammation or arthritis [39,40], is a well-known organic compound used as a fragrance ingredient in cosmetic products. Its use has been restricted in the European Union due to its irritant and allergenic properties. CinA is classified as an intermediate CS [41].

Our study showed that KC exposed to CinA did not lead to the same Nrf2-mediated antioxidant response regarding its concentration, since HO-1 and NQO-1, two main proteins involved in antioxidant activities, were differently expressed. Our results showed that the highest concentration of CinA led to a weak accumulation of Nrf2 with any expression of the HO-1 and NQO1 proteins and transcripts. Moreover, a correlation was observed between Nrf2 activation and the profile of the proinflammatory cytokines induced in KCs. The lowest concentration of CinA (100 µM) leading to the accumulation of Nrf2 was correlated with the absence of a proinflammatory response in KC (IL-1β, TNF-α IL-6, IL-8, and IL-23). CinA has been shown to induce CD54 and CD86 costimulatory molecules expression on THP1, a surrogate model of a dendritic cell model, and the production of proinflammatory factors such as IL-18 in KC [19,42]. On the other hand, CinA is efficient in mitigating a proinflammatory response in KC or monocytes/macrophages [43,44]. These results are in accordance with ours, showing that CinA has both pro- and anti-inflammatory effects in KC, depending on Nrf2 activation, which negatively regulates inflammation [15,25].

The observed opposite effect considering the inflammatory response of KCs exposed to CinA can be attributed to a more consistent cell death upon the 250-µM treatment, because Nrf2 whose function is primordial in the detoxification response is known to significantly improve cell survival upon chemical exposure [15]. Moreover, this exacerbated death of KC could also be responsible for an increased release of danger associated molecular patterns (DAMP) such as ATP or HMGB1 (high mobility group box 1), further activating the remaining living cells and amplifying the inflammatory response, also controlled by Nrf2.

Under proinflammatory stimulation such as Zymosan, the CinA concentration leading to Nrf2 induction was able to reduce the proinflammatory response, as already described in other models [39,44,45]. Thus, the anti-inflammatory properties of 100-µM CinA were retained. The role of Nrf2 in controlling the inflammatory state is also involved at the steady state in KC, since Nrf2 deficiency leads to an increase in the expression of proinflammatory cytokines such as IL-1β, IL-6, and IL-8. Therefore, counteracting the Nrf2 levels in KC may no longer control the inflammatory phenotype.

These results are consistent with the role of Nrf2 described as an essential regulator of inflammation by directly blocking the transcription of inflammatory cytokines such as IL-1β, IL-6, and TNF [25]. The difference in Nrf2 accumulation regarding CinA concentration could be related to Nrf2 stability and activation. P38 MAPK, which regulates Nrf2 activity [46], is highly activated in response to CinA 250 µM compared to CinA 100 µM (data not shown). This could explain the absence of Nrf2 activity in response to this high concentration of CinA.

The cellular metabolism and cellular metabolites themselves can control inflammation [47,48]. Since the modification of metabolism regulation or the metabolites level can alter immune cells’ functions, such as DC, CD4/CD8 T cells, or regulatory T cells [30,31,49], the metabolic modifications occurring in KC following exposure to CinA and the link with Nrf2 activity in the context of the proinflammation signal were assessed. Our results showed that cells treated with CinA 100 µM relied more on glycolysis to produce ATP and the absence of Nrf2. Wickersham et al. showed that blocking glycolysis in KC dampened their IL-1β production [50]. As already reported, CinA regulates cellular metabolism by modifying the expression of metabolic genes [51,52]. Our metabolomic analyses revealed dramatic changes in the metabolites concentration, underlying that seven metabolites were regulated in response to CinA 250 µM, a concentration that does not induce Nrf2. Among these metabolites, cystathionine and cis-aconitate, the precursors of itaconate, were more abundant in KC stimulated with CinA 100 μM and concentration-inducing Nrf2 than KC stimulated with CinA 100 μM or in Nrf2-deficient cells. Itaconate has also been shown to have anti-inflammatory properties by activating Nrf2 through the alkylation of Keap1 cysteine residues [53,54,55,56,57]. Lee et al. showed that cystathionine antagonizes the formaldehyde-induced upregulation of Matrix metalloproteinase-1 (MMP1), Prostaglandin E2 (PEG2), and chemokine (C-X-C motif) ligand 8 (CXCL8) in normal human KCs (NHKs) [58]. Our results suggest that Nrf2 may be a regulator of the cystathionine and cis-aconitate levels in KC in response to CinA.

Taken together, the fate of Nrf2 in the KC correlates with the anti- or proinflammatory state. The two metabolites, cystathionine and cis-aconitate, could participate in the Nrf2-dependent anti-inflammatory effect and could represent important biomarkers for characterizing the proinflammatory status of KCs [57]. Thus, Nrf2 is proposed to be involved in the metabolic responses regulating the inflammatory response to a CS.

## Data Availability

The data presented in this study are available in this manuscript.

## Supplementary Materials

The following are available online at https://www.mdpi.com/article/10.3390/antiox11030575/s1: Figure S1: CinA induces cytotoxicity on KERTr. KERTr were exposed to different concentrations (25–250 µM) of CinA or DMSO 0.1% as the vehicle control for 24 h, and cellular toxicity was determined by propidium iodide staining. Data represent the results of 4 independent experiments and are expressed as the mean ± SEM. * Represents the statistical difference between CinA and DMSO-treated cells. * *p*-value < 0.05 and ** *p*-value < 0.01 (Mann–Whitney test). Figure S2: Zymosan does not modify the CinA-induced antioxidant response. KERTr were exposed to different concentrations (25–100 µM) of CinA alone or at the same time as zymosan A (20 µg/mL) stimulation for 2 and 6 h. The mRNA level of *HO-1* and *NQO1* determined by RT-qPCR after 2 and 6 h of exposure to CinA ± zymosan. Data represent the results of 6 independent experiments and are expressed as the mean ± SEM. Figure S3: CinA-induced downregulation of IL-1β is concomitant to Nrf2 accumulation in KERTr. KERTr was exposed to 100 µM of CinA or DMSO 0.1% as the vehicle control from 30 min to 2 h. (A) The mRNA level of *IL-1β* determined by RT-qPCR. (B) Western blot and relative quantification of Nrf2. Data represent the results of 3–11 independent experiments and are expressed as the mean ± SEM. * Represents the statistical difference between CinA and vehicle-treated cells. * *p*-value < 0.05 and *** *p*-value < 0.001 (Mann–Whitney test). Figure S4: Invalidation of Nrf2 does not modify CinA-induced toxicity in KERTR. KERTR invalidated for Nrf2 (sh Nrf2) or not (sh ctrl) were exposed to different concentrations (10–250 µM) of CinA or DMSO 0.1% as the vehicle control for 24 h, and cellular toxicity was determined by propidium iodide staining. Data represent the results of 6 independent experiments and are expressed as the mean ± SEM. Figure S5: High concentrations of CinA and Nrf2 deficiency altered glycolysis in KERTr. KERTr were invalidated for Nrf2 by transduction with lentiviral particles to express a short-hairpin RNA targeting Nrf2 (sh Nrf2) or a scrambled short-hairpin (sh ctrl) as the control cells, and the cells were exposed to 100 or 250 µM of CinA for 1 h, followed by the seahorse analysis. (A) Extracellular acidification rate (ECAR) of sh ctrl cells exposed to CinA (100 or 250 µM) and DMSO 0.1% as the control. (B) ECAR of unstimulated sh ctrl and sh Nrf2 cells. (C) ECAR of sh ctrl and sh Nrf2 cells exposed to 100 µM of CinA and DMSO 0.1% as the control. Data represent the results of 1 experiment with 8 replicates. Table S1: Table of the identified metabolites by mass spectrometry in KERTr cells.

## Abbreviations

ACD: allergic contact dermatitis; ACOX, acyl-coenzyme A oxidase; ARE, antioxidant response element; ATP, adenosine triphosphate; CinA, Cinnamaldehyde; CPT, carnitine palmitoyltransferase; CS, contact sensitizers; CV, cell viability; DAMP, damage-associated molecular patterns; DC, dendritic cell; DMSO, dimethyl sulfoxide; ECAR, extracellular acidification rate; EGF, epidermal growth factor; GLUT1, glucose transporter type 1; HMGB1, high mobility group box-1; HO-1, heme oxygenase-1; HPLC, high-performance liquid chromatography; IL, interleukin; KC, keratinocytes; KEAP1, kelch-like ECH-associated protein 1; LC, Langerhans cells; MAPK, mitogen-activated protein kinase; MSD, meso scaled discovery technology; NAD+, nicotinamide adenine dinucleotide; NHK, normal human KC: NLRP3, NLR Family Pyrin Domain-Containing 3; NQO1, NAD(P)H quinone oxidoreductase 1; Nrf2, nuclear factor (erythroid-derived 2)-like 2; OCR, oxygen consumption rate; OXPHOS, oxidative phosphorylation; ROS, reactive oxygen species; Th17, IL-17-producing T helper; TLR2, toll-like receptor 2; TNCB, trinitrochlorobenzene; TNF-α, tumor necrosis factor α; UV, ultraviolet.
