# Supplementary material for: The Inflammatory Response in Human Keratinocytes Exposed to Cinnamaldehyde Is Regulated by Nrf2
Source: Antioxidants (Basel). 2022 Mar 17;11(3):575. doi: 10.3390/antiox11030575 (PMC8945052; doi:10.3390/antiox11030575)
Supplement: Supplementary file 1 [file antioxidants-11-00575-s001.zip › Supplementary Figures.pdf]

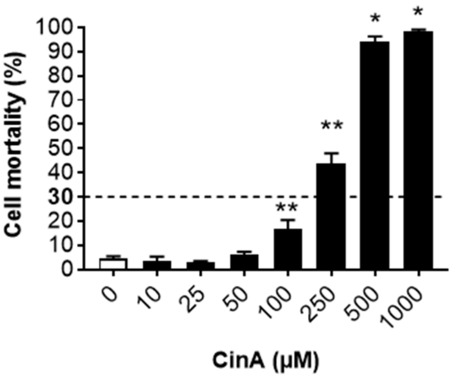

Figure S1. CinA induces cytotoxicity on KERTr. KERTr were exposed to different concentrations (25–250 µM) of CinA or DMSO 0.1% as the vehicle control for 24 h, and cellular toxicity was determined by propidium iodide staining. Data represent the results of 4 independent experiments and are expressed as the mean ± SEM. * Represents the statistical difference between CinA and DMSO-treated cells. * p-value < 0.05 and ** p-value < 0.01 (Mann–Whitney test).

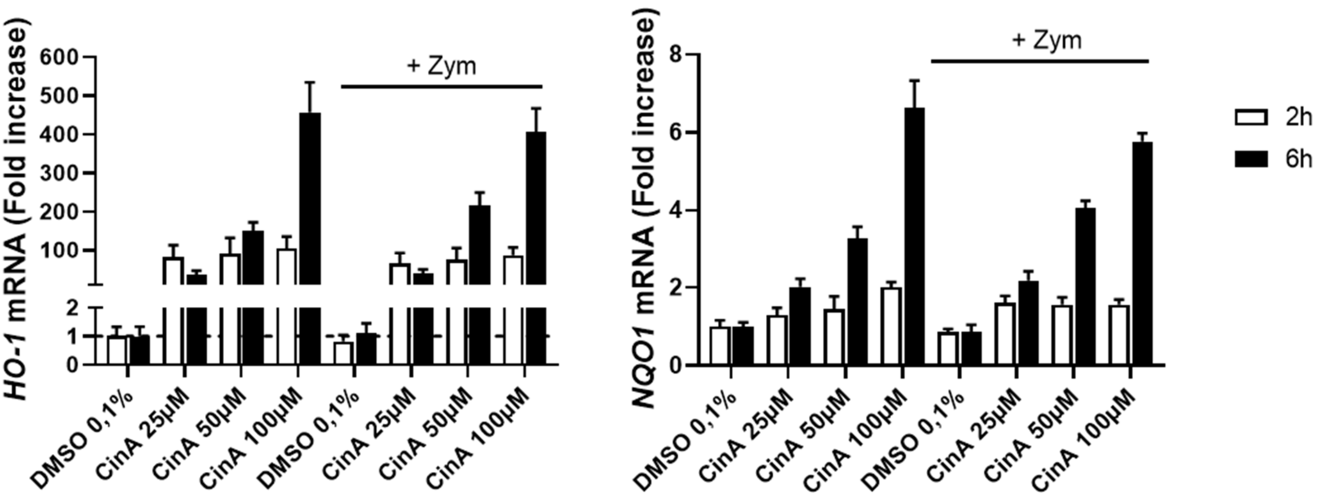

Figure S2. Zymosan does not modify the CinA-induced antioxidant response. KERTr were exposed to different concentrations (25–100 µM) of CinA alone or at the same time as zymosan A (20 µg/mL) stimulation for 2 and 6 h. The mRNA level of *HO-1* and *NQO1* determined by RT-qPCR after 2 and 6 h of exposure to CinA ± zymosan. Data represent the results of 6 independent experiments and are expressed as the mean ± SEM.

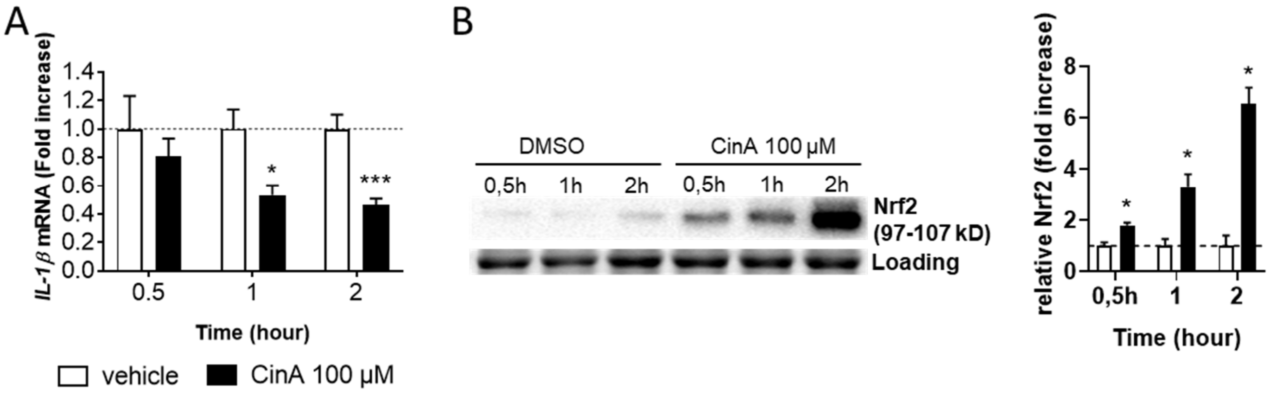

Figure S3. CinA-induced downregulation of IL-1β is concomitant to Nrf2 accumulation in KERTr. KERTr was exposed to 100 µM of CinA or DMSO 0.1% as the vehicle control from 30 min to 2 h. (A) The mRNA level of *IL-1β* determined by RT-qPCR. (B) Western blot and relative quantification of Nrf2. Data represent the results of 3–11 independent experiments and are expressed as the mean ± SEM. * Represents the statistical difference between CinA and vehicle-treated cells. * p-value < 0.05 and *** p-value < 0.001 (Mann–Whitney test).

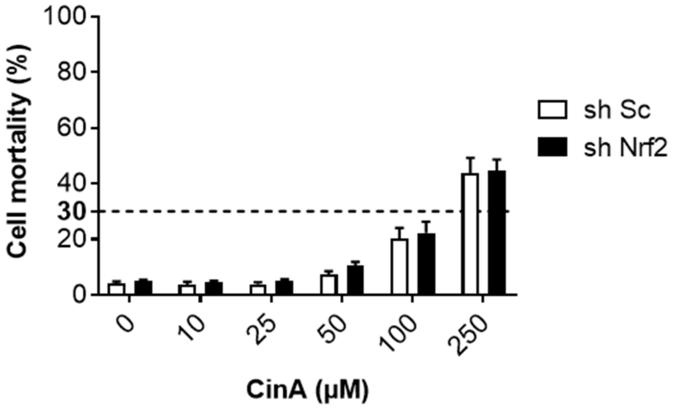

Figure S4. Invalidation of Nrf2 does not modify CinA-induced toxicity in KERTR. KERTR invalidated for Nrf2 (sh Nrf2) or not (sh ctrl) were exposed to different concentrations (10–250 µM) of CinA or DMSO 0.1% as the vehicle control for 24 h, and cellular toxicity was determined by propidium iodide staining. Data represent the results of 6 independent experiments and are expressed as the mean ± SEM.

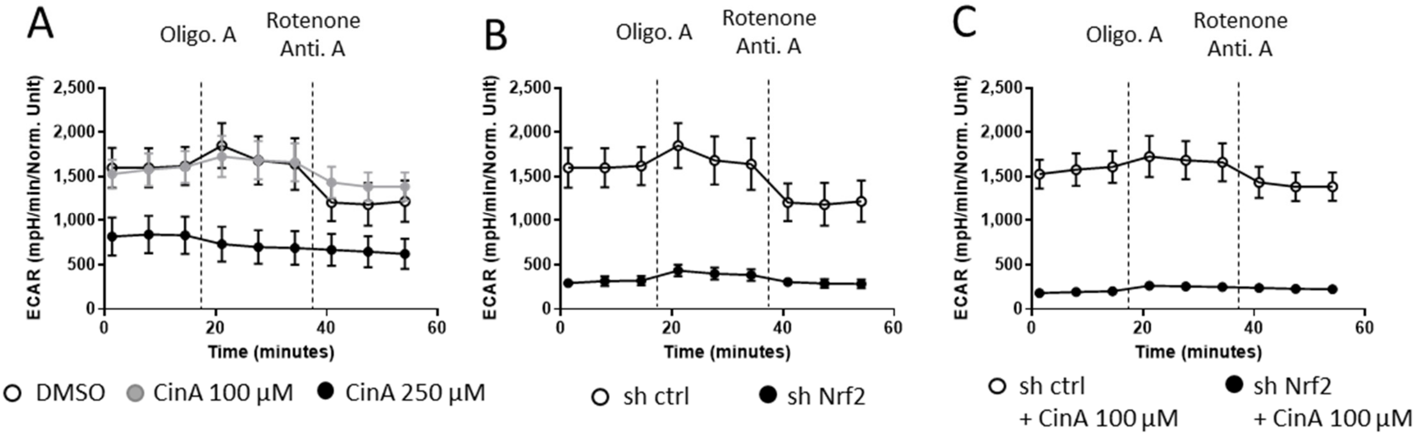

Figure S5. High concentrations of CinA and Nrf2 deficiency altered glycolysis in KERTr. KERTr were invalidated for Nrf2 by transduction with lentiviral particles to express a short-hairpin RNA targeting Nrf2 (sh Nrf2) or a scrambled short-hairpin (sh ctrl) as the control cells, and the cells were exposed to 100 or 250 µM of CinA for 1 h, followed by the seahorse analysis. (A) Extracellular acidification rate (ECAR) of sh ctrl cells exposed to CinA (100 or 250 µM) and DMSO 0.1% as the control. (B) ECAR of unstimulated sh ctrl and sh Nrf2 cells. (C) ECAR of sh ctrl and sh Nrf2 cells exposed to 100 µM of CinA and DMSO 0.1% as the control. Data represent the results of 1 experiment with 8 replicates.
